# Supplementary material for: Resolving the full spectrum of human genome variation using Linked-Reads
Source: Genome Res. 2019 Apr;29(4):635–45. doi: 10.1101/gr.234443.118 (PMC6442396; doi:10.1101/gr.234443.118)
Supplement: Supplemental Material [file supp_gr.234443.118_Supplemental_longranger-2.2.zip › longranger-2.2.tar.bz2!/lr2.2-public/lib/stan/cmdstan/stan/doc/api/html/fwd_2operators_2operator__subtraction_8hpp_source.html]

Stan: src/stan/agrad/fwd/operators/operator\_subtraction.hpp Source File


|  |  |
| --- | --- |
|  | Stan  2.5.0  probability, sampling & optimization |


- Main Page
- Related Pages
- Namespaces
- Classes
- Files

- File List
- File Members

All Classes Namespaces Files Functions Variables Typedefs Enumerations Enumerator Friends Macros Pages

- src
- stan
- agrad
- fwd
- operators

operator\_subtraction.hpp

Go to the documentation of this file.

1 #ifndef STAN\_\_AGRAD\_\_FWD\_\_OPERATORS\_\_OPERATOR\_SUBTRACTION\_HPP

2 #define STAN\_\_AGRAD\_\_FWD\_\_OPERATORS\_\_OPERATOR\_SUBTRACTION\_HPP

3

4 #include <stan/agrad/fwd/fvar.hpp>

5 #include <stan/meta/traits.hpp>

6

7 namespace stan {

8

9  namespace agrad {

10

11  template <typename T>

12  inline

13  fvar<T>

14  operator-(const fvar<T>& x1, const fvar<T>& x2) {

15  return fvar<T>(x1.val\_ - x2.val\_, x1.d\_ - x2.d\_);

16  }

17

18  template <typename T>

19  inline

20  fvar<T>

21  operator-(const double x1, const fvar<T>& x2) {

22  return fvar<T>(x1 - x2.val\_, -x2.d\_);

23  }

24

25  template <typename T>

26  inline

27  fvar<T>

28  operator-(const fvar<T>& x1, const double x2) {

29  return fvar<T>(x1.val\_ - x2, x1.d\_);

30  }

31  }

32 }

33 #endif

stan::agrad::fvar

**Definition:** fvar.hpp:13

stan::agrad::operator-

fvar< T > operator-(const fvar< T > &x1, const fvar< T > &x2)

**Definition:** operator\_subtraction.hpp:14

traits.hpp

fvar.hpp

stan::agrad::fvar::d\_

T d\_

**Definition:** fvar.hpp:16

stan::agrad::fvar::val\_

T val\_

**Definition:** fvar.hpp:15


---

[ Stan Home Page ]

*© 2011–2014,
Stan Development Team.*
